# A Rare Case of Blastic Plasmacytoid Dendritic Cell Neoplasm Occurred in Postchemotherapy of Breast Cancer

**DOI:** 10.1155/2023/7573037

**Published:** 2023-07-06

**Authors:** Jiankun Tong, Sergei Aksenov, Beth M. Siegel, Lihong Wei, William H. Rodgers

**Affiliations:** ^1^Department of Pathology, New York Presbyterian Queens, 56-45 Main Street, Flushing, NY 11355, USA; ^2^Department of Surgery, Section of Breast Surgery, New York Presbyterian Queens, 58-04 Main Street, Flushing, NY 11355, USA; ^3^Consultant Hematology & Medical Oncology, Queens Medical Associates, 176-60 Union Tpke # 360, Fresh Meadows, NY 11366, USA; ^4^Weill Cornell Medical College, 525 East 68th Street, Box 130, New York, NY 10065, USA

## Abstract

Blastic plasmacytoid dendritic cell neoplasm (BPDCN) is a rare and highly aggressive hematologic malignancy that arises from plasmacytoid dendritic cells. BPDCN typically presents with skin lesions and may involve peripheral blood, bone marrow, lymph nodes, or extranodal sites. It usually arises de novo, and some BPDCN cases are associated with or develop into myeloid neoplasms. Here, we report a case of a 57-year-old female presenting with cervical lymphadenopathy and skin rashes during the COVID-19 pandemic in 2021 following multiple types of postmastectomy therapy for breast cancer. The patient was ultimately diagnosed with BPCDN by lymph node biopsy. To the best of our knowledge, this is the first case report of BPDCN occurring postchemotherapy of breast cancer.

## 1. Introduction

Blastic plasmacytoid dendritic cell neoplasm (BPDCN) is a rare and highly aggressive hematologic malignant neoplasm derived from the precursors of plasmacytoid dendritic cells. BPDCN predominantly occurs in the elderly, although it can occur at any age, including in children [1–3]. Prevalence is approximately 3.3 times higher in males compared to females [1]. BPDCN involves multiple sites and organs, with the highest preference for skin (in 64–100% cases), followed by bone marrow and peripheral blood (80–90% cases), lymph node (40–50% cases), and spleen (20% cases) [4]. Less frequently, it was also shown to affect breast, brain and central nervous system, liver, lung, paranasal sinus, testis, and other sites [1, 5–9].

The etiology of BPDCN remains unknown [1]. There is no known association with EBV [1, 3]. It usually arises de novo; though some BPDCN cases (10–20%) are associated with, or develop into other myeloid neoplasms, most commonly chronic myelomonocytic leukemia (CMML), myelodysplastic syndrome (MDS), or acute myeloid leukemia (AML) [1]. Association with myeloid neoplasms suggests related pathogenesis [1].

The development of various therapy-related myeloid neoplasms (t-MN) following breast cancer chemotherapy has been confirmed [10–12]. However, we have not come across any reports specifically describing BPDCN after chemotherapy of breast cancer. Here, we report a case of 57-year-old female presenting with lymphadenopathy on the left and right sides of her neck, skin rashes, pancytopenia, and blasts in the peripheral blood. The patient was diagnosed with BPDCN six years following mastectomy and chemotherapy for breast cancer. We believe this is the first case report of BPDCN occurring postchemotherapy of breast cancer.

## 2. Case Presentation

**Figure 1:**
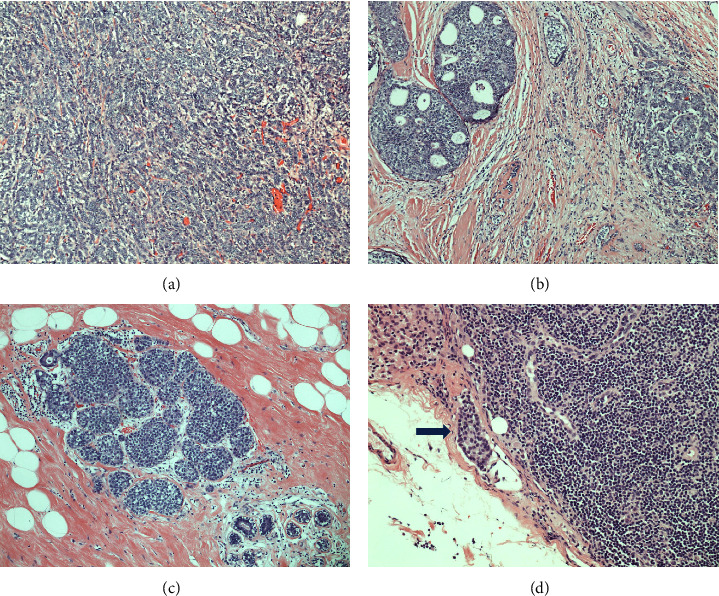
Invasive ductal carcinoma (IDC), moderately-differentiated ((a) x100). Ductal carcinoma in situ (DCIS), intermediated nuclear grade, cribriform type ((b) x100). Lobular carcinoma in situ (LCIS), classic type ((c) x100). Metastatic carcinoma in the lymph node, arrow indicating focus of metastatic carcinoma ((d) x100).

The patient is a 57-year-old Asian female who was diagnosed with cancer of the right breast in July 2015. She first presented with a palpable 2.0 cm nodule on her right breast. Mammogram taken in July 2015 showed a nodule suspicious for malignancy. Ultrasound showed suspicious regular palpable 1.7 cm mass with adjacent 0.9 cm irregular nodule in the 2 : 30–3 : 00 right breast. The patient underwent a surgical core biopsy in July 2015, which showed invasive ductal carcinoma, moderately differentiated (tubule formation score 3/3, nuclear grade score 2/3, and mitoses score 2/3). The invasive ductal carcinoma was strongly positive for estrogen receptor (ER) (>95%) and progesterone receptor (PR) (>95%). HER-2 by immunohistochemistry was equivocal (score 2+) and negative by fluorescence in situ hybridization (FISH). The Ki-67 proliferation index was 5–10%. The patient underwent right mastectomy with axillary sentinel lymph node dissection in September 2015, and the final pathology showed invasive ductal carcinoma, moderately-differentiated (grade 2) (Figure 1(a)), with solid papillary features and necrosis, measuring 3.5 cm in the greatest dimension, with clear surgical margins. It was associated with ductal carcinoma in situ (DCIS) (Figure 1(b)) and lobular carcinoma in situ (LCIS) (Figure 1(c)). Extensive lymphovascular invasion (LVI) and perineural invasion (PNI) were also identified. One of two right axillary sentinel lymph nodes was positive for micrometastatic carcinoma (Figure 1(d)). The final pathological stage was pT2N1mi with clear surgical margins.

Subsequently, the patient underwent AC chemotherapy with adriamycin and cyclophosphamide, every 2 weeks for 4 cycles between August 2015 and January 2016. This was followed by Taxol (paclitaxel) weekly for a total of 12 treatments (last treatment was done in mid-April 2016). She was treated further with endocrine therapy (tamoxifen) from April 2016 to October 2019 and then changed to aromatase inhibitor with anastrozole after menopause.

The patient was followed up regularly and did not show any complications until May 2021, when she presented with palpable cervical lymphadenopathy and skin lesions. Notably, this also coincided with receiving her second COVID-19 vaccine. The lymphadenopathy developed 1-2 weeks after she received the second dose of COVID-19 vaccine. Neck ultrasound performed in May 2021 showed multiple mildly enlarged and prominent subcentimeter lymph nodes throughout the neck bilaterally and within both parotid glands; the impression on ultrasound is likely reactive in the setting of recent vaccination.

The patient was evaluated by an ENT physician who performed a fine needle aspiration on left neck lymph node in June 2021. The pathology showed a heterogeneous lymphoid population, favoring a reactive lymph node.

Her repeated computed tomography (CT) on neck in June 2021 revealed an extensive lymphadenopathy throughout the neck and mediastinum. Although COVID-19 vaccination can result in reactive lymphadenopathy, this extensive lymphadenopathy bilaterally would be highly atypical and the findings can be related to malignancy such as lymphoma. The patient was subsequently hospitalized to our hospital and underwent further work up including lymph node biopsy.

## 3. Laboratory Studies and CT Studies

**Figure 2:**
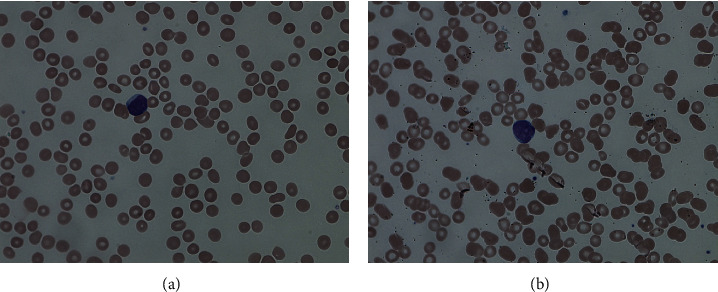
The peripheral blood showed medium-sized cells that have scanty cytoplasm, fine chromatin, and small nucleoli, resembling lymphoid blasts ((a) and (b), x400).

Laboratory analysis revealed mild leukopenia (WBC 3.36 K/L), anemia (HB 7.8 g/L), and thrombocytopenia (95 K/UL) with an abnormal differential showing atypical lymphocytes/blasts 21%, neutrophils 35%, bands 10%, lymphocytes 21%, monocytes 9%, and metamyelocytes 4%. The peripheral blood smear showed the atypical lymphoid cells to be medium-sized, and with scanty cytoplasm, fine chromatin, and small nucleoli, resembling lymphoid blasts (Figures 2(a) and 2(b)). COVID-19 testing (SARS-CoV-2 NAAT by real-time PCR of nucleic acid amplification) and CMV testing were not detected. Cytogenetics showed a normal female karyotype observed in 20 analyzed metaphase cells. No numerical or structural abnormalities of clinical significance were found in these cells. FLT3 mutation by PCR is negative for both internal tandem duplication (ITD) and tyrosine kinase domain (TKD). Epstein–Barr Virus (EBV) serology signified a past infection. HIV testing was negative. CT scan showed bilateral diffuse cervical lymphadenopathy along with bilateral intraparotid glands, mediastina, and left axillary lymphadenopathy.

## 4. Diagnosis

**Figure 3:**
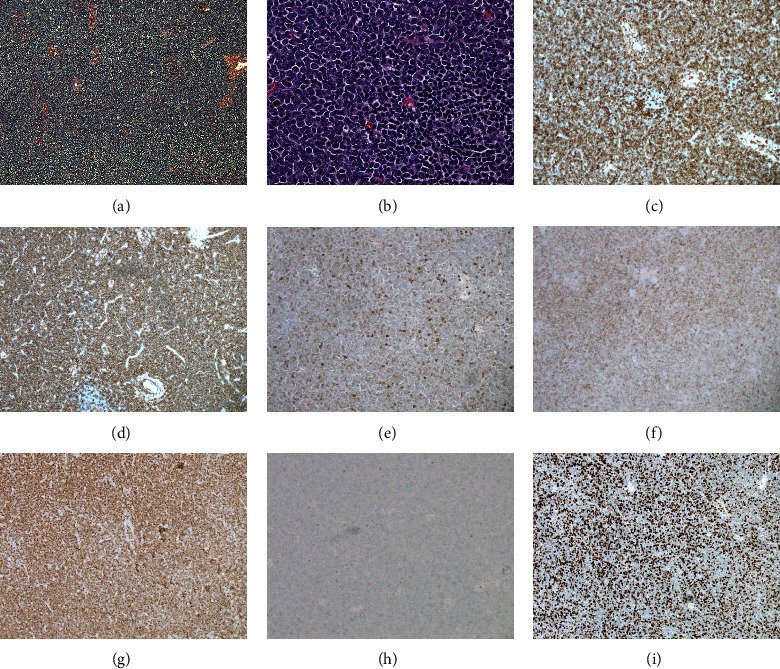
The lymph node architecture is completely effaced by a proliferation of the neoplastic cells ((a) x100), which are medium-sized, with fine chromatin, scanty cytoplasm, and indistinct nucleoli ((b) x400). The tumor cells show immunoreactivity for CD4 (c), CD43 (d), CD56 (e), CD123 (f), and TdT (g), while they are negative for MPO (h). The Ki-67 proliferation index is markedly elevated (50–60%) (i).

An excisional lymph node biopsy on the right neck was performed. The lymph node architecture was completely effaced by a diffuse proliferation of medium-sized atypical hematopoietic cells with a scant amount of cytoplasm, a round, oval, coffee-bean shaped, or irregular nuclei with indistinct nucleoli (Figures 3(a) and 3(b)). Mitotic figures were frequently identified. By immunohistochemistry, the atypical hematopoietic cells showed immunoreactivity for BCL-2, CD4 (Figure 3(c)), CD33, CD43 (Figure 3(d)), CD45, CD56 (partial and weak) (Figure 3(e)), CD68 (perinuclear dot), CD123 (weak) (Figure 3(f)), and TdT (Figure 3(g)), while they were negative for BCL-1, BCL-6, CD3, CD5, CD8, CD10, CD15, CD20, CD23, CD30, CD34, CD138, CD163, MUM-1, MPO (Figure 3(h)), and PAX-5. CD99 and CD117 are equivocal. P53 shows a wild type staining pattern. The Ki-67 proliferation index was approximately 50–60% (Figure 3(i)). EBER by in situ hybridization was negative. The morphology and immunostaining profile were diagnostic of blastic plasmacytoid dendritic cell neoplasm (BPDCN), and flow cytometry performed on the peripheral blood later confirmed the diagnosis of BPDCN. The patient was transferred to an advanced leukemia and lymphoma center for a clinical trial with Tagraxofusp. Later, the patient received a stem cell transplantation, and she is currently in remission.

## 5. Discussion

BPDCN is a rare aggressive hematopoietic neoplasm derived from plasmacytoid dendritic precursor cells (type I interferon-producing cells or plasmacytoid monocytes). Since this neoplasm was first described by Adachi M's group in 1994 [13], BPDCN has been referred to by various names, including agranular CD4+ natural killer (NK) leukemia [14], CD4+/CD56+ hematodermic neoplasm [2, 15], and blastic NK lymphoma [16]. In 2008, the WHO placed this rare neoplasm in the category of AML and related precursor neoplasms [17] following the realization that BPDCN results from the clonal proliferation of immature plasmacytoid dendritic cells (PDC) [18]. In 2016, the WHO classified BPDCN as a distinct neoplastic entity [1]. In normal situations, the PDCs are an essential part of the innate adaptive immunoresponse [19, 20]. These cells respond to bacterial and viral infections and other pathogens by producing alpha-interferon; hence, they are also known as alpha-interferon producing cells or plasmacytoid monocytes. Proliferation of the normal PDCs can occur in autoimmune disorders as well [19, 20].

### 5.1. Histologic and Immunophenotypic Features

Microscopically, BPDCN is characterized by a diffuse, monomorphous proliferation of small to medium-sized hematopoietic cells resembling either lymphocytes or myeloid blasts. Immunohistochemically, the tumor cells usually express CD4, CD43, CD45RA, and CD56, as well as the PDC-associated antigens CD123, CD303, and TCL1A, while they are negative for CD3, CD13, CD16, CD19, CD20, CD34, lysozyme, myeloperoxidase (MPO), and PAX-5. In addition, they are variably positive for BCL-2, BCL-6, CD33, CD68, CD117, S100, TCF4, and TdT. The proliferation index as assessed by Ki-67 is variable from 20% to 80%. The absence of lineage-associated antigens, together with positivity for CD4, CD45RA, CD56, and CD123 is considered as a unique immunophenotype virtually pathognomonic of BPDCN [21]. CD303 positivity has the highest diagnostic score within a panel of markers used for BPDCN identification [22]. Genetically, T-cell and B-cell receptor gene mutations are usually germline. Specific chromosomal aberrations are absent, but complex karyotypes are commonly present [1].

### 5.2. Diagnosis and Differential Diagnosis

The diagnosis of BPDCN is based on the clinical manifestation, histology/morphology, immunohistochemistry, and flow cytometry. Because it shows some similar morphological and immunohistochemical features to other malignant neoplasms, extensive immunohistochemistry, flow cytometry, and/or genetic analysis are essential for a definite diagnosis of BPDCN. The differential diagnosis predominantly includes hematopoietic neoplasms and nonhematopoietic neoplasms. The former consists mainly of AML with monocytic differentiation, T-lymphoblastic leukemia/lymphoma or early T-cell precursor lymphoblastic leukemia/lymphoma, extranodal NK-T-cell lymphoma, mature T-cell lymphoma, myeloid sarcoma, histiocytic sarcoma, and others. The latter includes malignant melanoma, poorly-differentiated carcinoma, and high grade/undifferentiated sarcoma.

### 5.3. Therapy-Related Myeloid Neoplasms and Postchemotherapy of Solid Tumors

Therapy-related myeloid neoplasms (t-MN) are thought to be the consequence of mutational events induced by cytotoxic therapy. Cytotoxic agents implicated in therapy-related myeloid neoplasm include alkylating agents, ionizing radiation therapy, topoisomerase II inhibitors, and others [1]. The alkylating agents demonstrated and implicated in t-MN include melphalan, cyclophosphamine, nitrogen mustard, chlorambucil, busulfan, carboplatin, cisplatin, decarbazine, procarbazine, carmustine, mitomycin C, thiotepa, lomustine, ifosfamide, and temozolomide [1, 11, 23]. They have been known for many years to induce t-MN. Studies have demonstrated chromosomal deletions involving the 5q and 7q regions, as well as complex karyotypes and mutation or loss p53 commonly present in these patients [24–27]. These chromosomal losses lead to several gene defects that are involved in haematopoiesis and may trigger the progression to t-MN [28, 29].

Topoisomerase II inhibitors linked to and implicated in t-MN include etoposide, teniposide, doxorubicin, daunorubicin, mitoxantrone, amsacrine, and actinomycin [1, 11, 23]. Topoisomerase II inhibitors hinder DNA topoisomerases. DNA topoisomerases are enzymes critical for cellular function as they regulate DNA winding through removal of knots and tangles [30]. They execute important cellular activities such as ensuring DNA stability during replication. Inhibited function can cause cell damage by trapping the enzymes in covalent complexes on the DNA [30]. The other agents used for the treatment of solid tumors (not implicated here) are antimetabolites and antitubulin agents which include vincristine, vinblastine, vindesine, paclitaxel, and docetaxel [1].

The most common cohort of t-MN occurs 5–10 years after exposure to alkylating agents and ionizing radiation [1]. The second t-MN subset accounts for 20–30% of cases, has a shorter latency period of 1–5 years, and is associated with treatment agents that interact with DNA topoisomerase II (e.g., topoisomerase II inhibitors) [1]. The site of involvement is predominantly peripheral blood and bone marrow. In t-MN patients, about 70% have been treated for a solid tumor and 30% for a hematological neoplasm, with breast cancer making up the largest numbers of cases [31–33]. The development of therapy-related myeloid neoplasm (t-MN) after chemotherapy of breast cancer has been confirmed [31–33]. In 20–30% of t-MN cases, the first manifestation is overt acute leukemia, without a preceding myelodysplastic phase [1].

### 5.4. Is BPDCN COVID-19 Vaccine-Related?

COVID-19 vaccines are commonly administered intramuscularly to the arm/deltoid muscle. An association between vaccine administration and the development of ipsilateral axillary and supraclavicular lymphadenopathy has been reported [34, 35]. The incidence of lymphadenopathy varies. One review showed pooled incidence of clinically detected lymphadenopathy after COVID-19 vaccination was 0.4% [35]. A recently published retrospective case series analyzed the mammograms of patients with a history of administration of at least 1 dose of a COVID-19 vaccine within the previous 90 days and identified 23 cases of axillary adenopathy (3%), which is higher than reported rates of axillary lymphadenopathy in otherwise normal mammography (0.02–0.04%) [34, 36]. The incidence of lymphadenopathy was found to be higher in the first 2 weeks following the vaccination, and it usually resolves within 3–6 weeks [34–36]. If lymphadenopathy persists for more than 6 weeks, or becomes worse, an appropriate management strategy should be taken. In our case, the patient developed multiple enlarged lymph nodes throughout the neck bilaterally, within both parotid glands, and mediastinum 1-2 weeks after the second dose of COVID-19 vaccine. The possibility that receiving a second dose of the COVID-19 vaccine initiated or aggravated the lymphadenopathy cannot be disproved. However, we do not have solid evidence to suggest that the vaccine directly caused or resulted in lymphadenopathy. Presently, no published literature provides direct evidence to support that COVID-19 can induce hematological malignant neoplasms.

### 5.5. Is BPDCN Therapy-Related or Does It Arise De Novo?

In our case, several interesting phenomena were observed. First, the patient had a history of invasive and metastatic breast cancer, and she received chemotherapy including alkylating agent (cyclophosphamide), topoisomerase II inhibitor (doxorubicin/adriamycin), antitubulin agent (paclitaxel), estrogen receptor antagonist (tamoxifen), and aromatase inhibitors (anastrozole). Second, the patient developed BPDCN six years of postchemotherapy, which falls in the range of about 5–10 years after exposure to alkylating agents [1]. Third, the involvement is not local or limited to lymph nodes, but is systemic and extensive, including bone marrow, peripheral blood, and multiple lymph nodes (bilateral diffuse cervical lymphadenopathy along with bilateral intraparotid glands, mediastina, and left axillary lymphadenopathy). Fourth, the patient had no history of myelodysplastic syndrome or related hematopoietic neoplasms before, except breast carcinoma. Fifth, cytogenetics showed a normal female karyotype without any numerical and structural abnormalities. On the one hand, several related factors observed in our patient's data (presented above) can suggest causality and raise the possibility that BPDCN here may be a therapy-related occurrence. On the other hand, no direct evidence to support such claim exists. Due to the complexity of possible interactive effects of treatment agents, broad timeline, potential interference of additional unknown factors and random process, and the sheer rarity BPDCN in general, the genuine causes cannot be accurately determined.

In conclusion, we reported what we believe is the only case to date of BPDCN occurring postmastectomy and postchemotherapy of breast cancer. The absence of similar case reports and complexity of etiology preclude us from making any conclusions regarding potential causality or relationship between breast cancer chemotherapy and BPDCN. However, given that numerous reports identify a link between chemotherapy agents and other types of t-MNs, we cannot entirely dismiss this possibility. While de novo manifestation remains entirely plausible, we would like to encourage medical practitioners to at least consider BPDCN as a potential risk factor of breast cancer chemotherapy, albeit highly rare and unlikely.

## Data Availability

The data used to support the findings of this study are included within the article.
